# Treatment of median arcuate ligament syndrome: outcome of laparoscopic approach

**DOI:** 10.1590/0102-672020190001e1495

**Published:** 2020-05-18

**Authors:** Julio Cezar Uili COELHO, Andréa Virmond El HOSNI, Christiano MarloPaggi CLAUS, Yan Sacha Hass AGUILERA, Gisele Pitrowsk ABOT, Alexandre Teixeira Coutinho de FREITAS, Marco Aurélio Raeder da COSTA

**Affiliations:** 1 Department ofSurgery, Hospital Nossa Senhora das Graças, Curitiba, PR, Brazil

**Keywords:** Median arcuate ligament syndrome, Celiac axis compression syndrome, Celiac artery, Laparoscopic surgery, Síndrome do ligamento arqueado mediano, Síndrome da compressão do tronco celíaco, Artéria celíaca, Cirurgia laparoscópica

## Abstract

***Background*:**

Median arcuate ligament syndrome(MALS) is a rare condition thatmay cause significant clinical manifestations, including abdominal pain and weight loss. Its diagnosis may be difficult and very often delayed. The laparoscopic approach became the standard treatment of MALS.

***Aim*:**

To assess the outcome of laparoscopic treatment in patients with MALS.

***Method*:**

The data of sixpatients with MALS who were subjected to laparoscopic sectioning of the median arcuate ligament were retrospectively reviewed.The following data were evaluated: age, gender, clinical and diagnostic tests findings, ASA score, operative findings and complications, postoperative complications and mortality, hospital stay duration, and hospital readmission.The diagnosis of MALS was established by CT angiography and/or MR angiography.

***Results*:**

There were four (66.7%) women and two (33.3%) men aged from 32 to 60 years. The main symptoms were epigastric pain (100%) and weight loss (66.7%). The findings of high-grade stenosis of the proximal celiac axis and poststenotic dilation confirmed on angiography confirmed the diagnosis in all patients. Surgical procedure was uneventful in all patients. The only postoperative complication was urinary retention that occurred in a male. At three-month follow-up, all patients were asymptomatic.

***Conclusion*:**

Laparoscopic treatment of MALS is safe and effective in relieving the clinical manifestations of patients.

## Graphical Abstract

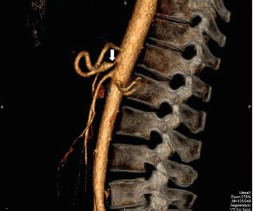

## RESUMO

***Racional*:**

A síndrome do ligamento arqueado mediano (SLAM) é condição rara que pode causar manifestações clínicas significativas, incluindo dor abdominal e perda de peso. Seu diagnóstico pode ser difícil e muitas vezes estabelecido tardiamente. A abordagem laparoscópica tornou-se o tratamento padrão para ela.

***Objetivo*:**

Avaliar o resultado do tratamento laparoscópico em pacientes com SLAM.

***Método*:**

Os dados de seis pacientes com SLAM submetidos a ressecção laparoscópica do ligamento arqueado mediano foram revisados ​​retrospectivamente. Os seguintes dados avaliados foram: idade, gênero, resultados dosexames clínicos e complementares, escore ASA, achados e complicações operatórias, complicações e mortalidade pós-operatórias, tempo de internação e readmissão hospitalar. O diagnóstico de SLAM foi estabelecido por angiotomografia e/ou angiorressonância.

Havia quatro (66,7%) mulheres e dois (33,3%) homens com idades entre 32 e 60 anos. Os principais sintomas foram dor epigástrica (100%) e perda de peso (66,7%). Os achados de estenose de alto grau do tronco celíaco proximal e dilatação pós-estenótica observados na angiografia confirmaram o diagnóstico em todos os pacientes. O procedimento cirúrgico transcorreu sem intercorrências em todos os pacientes. A única complicação pós-operatória foi retenção urinária, que ocorreu em um homem. No seguimento de três meses, todos os pacientes estavam assintomáticos.

***Conclusão*:**

O tratamento laparoscópico da SLAM é seguro e eficaz no alívio das manifestações clínicas dos pacientes.

## Graphical Abstract

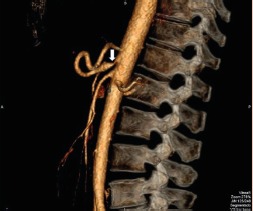

## INTRODUCTION

Median arcuate ligament syndrome(MALS) - also namedDunbar syndrome or celiac axis compression syndrome - is due to compression of the celiac axis and/or celiac ganglion by the median arcuate ligament of the diaphragm^1^ ^,^ ^3^ ^,^ ^15^ ^,^ ^20^. It was first described by Harjola^6^ in 1963 who reported resolution of postprandial upper abdominal pain and epigastric bruit in a man following surgical decompression of the celiac axis due to a fibrous celiac ganglion.Dunbar et al^4^ in 1965 reported a series of 15 patients with abdominal angina due to partial celiac artery compression by the median arcuate ligament. Thirteen of these 15 patients underwent surgical decompression of the celiac artery and had relief of abdominal pain.

Although the incidence of median arcuate ligament syndrome is unknown, case and series reportsit have increased in the last years, possibly due to widespread use of imaging exams, such as computed tomography and magnetic resonance^2^ ^,^ ^5^ ^,^ ^7^ ^-^ ^10^. It may cause various clinicalsymptoms such as abdominal pain, weight loss, nausea and vomiting, and diarrhea. The pain isvariable and it may be mainly postprandial and alleviated by leaning the body forward^11^ ^-^ ^14^.In athletes, the pain may be exercise-induced rather than postprandial^7^. Epigastric bruit may be recognized during physical examination. Severe complications such as ruptured pseudo-aneurysm of the inferior pancreatic-duodenal artery have also been reported^15^ ^,^ ^21^. The most usual treatment of this condition is surgical decompression of the celiac axis by sectioning the median arcuate ligament^5^ ^,^ ^19^ ^,^ ^22^. Since the first release of the celiac axis through laparoscopy by Roayaie et al.^17^ in 2000, this access became the standard treatment of MALS.

Our objective was to evaluate the outcome of laparoscopic treatment of patients with MALS in our hospital. 

## METHODS

This prospective observational study included data from six patients with MALS who were subjected to laparoscopic sectioning of the median arcuate ligament at Hospital Nossa Senhora das Graças, Curitiba,PR, Brazil, from October 2014 through June 2019.The study was approved by the Investigation Ethics Committee of the Hospital de Clínicas of the Federal University of Parana, Curitiba, Brazil (Protocol approval number 3.037.086).Informed consentwas waived due to the retrospective study design and collection of readily available clinical data.

### Data collection

The following data were obtained: age, gender, clinical and diagnostic test findings, American Society of Anesthesiologists score (ASA), operative findings and complications, postoperative complications and mortality, hospital stay duration, and hospital readmission. Data were obtained retrospectively from electronic medical records and study protocols. Values were expressed as mean±SD (standarddeviation).

The diagnosis of MALS was confirmed in all patients by CT angiography and/or MR angiography. Other causes of abdominal pain were excluded with extensive medical evaluation, including laboratory tests, electrocardiogram, abdominal ultrasonography, small bowel radiographic study, upper gastrointestinal endoscopy, and colonoscopy. 

### Surgical procedure

Under general anesthesia, the patient was placed in supine position in reverseTrendelenburg position with the legs abducted and supported on cushioned spreader bars. The surgeon stood between the patient’s legs. A temporary nasogastric tube was inserted. Thromboembolism prophylaxiswith enoxaparin sodium 40 mg was administered subcutaneouslyat the anesthesia induction in patients ≥40years old, and in patients with obesity or historyof previous thromboembolism. Antibiotic prophylaxis was not used. Immediately prior to wound incisions, all layers of the abdominal wall of the wounds were infiltrated with local anesthetic (bupivacaine 0.5%). The patients received a single intra-operative dose of intravenous parocoxib sodium 40 mg, tramadol hydrochloride 100 mg, and dipyrone 2 g for analgesia. A single dose of 4 mg of ondansetron was administered intravenously prior to completion of the procedure to prevent postoperative nauseas and vomiting. 

The operation was performed through five trocars inserted in the upper abdomen, similar to that of Nissen-Rosetti procedure. A camera port was inserted on the midline at about 5 cm above the umbilicus. Four additional trocars were inserted under direct vision into the right and left subcostal areas, left flank, and subxiphoid position. 

A retractor was inserted into the subxiphoid trocar to elevate the left lobe of the liver medially and the stomach was retracted to the patient’s left side with a Babcock clamp.LigaSure™ Maryland jaw device (Medtronics, Minneapolis, MN, USA) was employed for tissue dissection and hemostasis. 

After gastrohepatic ligamentsectioning, the common hepatic artery and the left gastric artery were dissectedand isolated with a vessel loop for retraction. The arteries were followed to the origin of the celiac trunk. There was no need to dissect the splenic artery.

**FIGURE 1:**
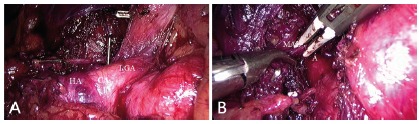
Laparoscopic treatment of median arcuate ligament syndrome: A)common hepatic artery (HA) and left gastric artery (GA) are isolated and dissected andthe celiac axis (CA) is exposed; B) section of the median arcuate ligament (MAL) with LigaSureMaryland jaw device is shown and exposure of the anterior wall of the aorta (A) is also depicted.

The right crus was dissected and the median arcuate ligament was identified and divided by energy device and/or a hook cautery (Figure 1). The anterior surface of the aorta was exposed for about 3 cm. The celiac trunk was completely skeletonized.All fibrotic tissue and nerve plexus overlying the celiac axis were resected.Undue dissection of the esophagus hiatus was not performed in order to avoid postoperative gastroesophageal reflux. Intraoperative ultrasonography was not employed to assess celiac artery flow after decompression.

### Follow-up

Patients returned for ambulatory follow-up on the 7^th^ day, and one and three months after operation. Follow-up was extended as needed. Control CT angiography or MR angiography was performed at the 3^rd^ PO month.

## RESULTS

There were four(66.7%) women and two (33.3%) men aged from 32 to 60 years, with a mean age of 43.3±12.8 years (mean±SD). Clinical manifestations lasted from eight months to three years. Intermittent epigastric pain was referred by all patients. Four(66.7%) patients complained that the pain was postprandial and relieved with fasting. These four(66.7%) had weight loss of 3-6 kg, with a mean of 4.8±1.4 kg. All denied nausea, vomiting and diarrhea. Physical examination was normal in all patients (Table 1).

**TABLE 1:** Demographic and clinical aspects of patients

Aspects	n	%
Number	6	
Age (years)		
Mean±SD	43.3±12.8	
Range		32-60
Gender		
Female	4	66.7
Male	2	33.3
Clinical Presentation		
Epigastric pain	6	100
Postprandial pain	4	66.7
Weight loss	4	66.7
ASA score		
I	4	66.7
II	2	33.3
Prior abdominal surgery	1	16.7
CT Angiography celiac axis		
High-grade stenosis	6	100
Poststenotic dilation	6	100

*ASA=American Society of Anesthesiologists

**FIGURE 2:**
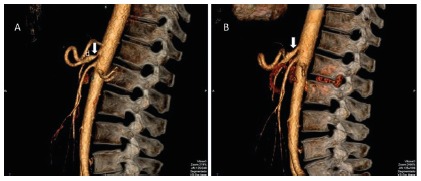
A) Computed tomographic angiography of the abdomen demonstrating severe stenosis of the proximal segment of the celiac axis (arrow) caused by extrinsic compression of the median arcuate on left side, and poststenotic dilation is also depicted with letter “d”;B) normal celiac axis (arrow) is shown after section of the median arcuate ligament on the right side.

The diagnosis of MALS was confirmed in all patients by CT angiography and/or MR angiography that showed high-grade stenosis of the anterior wall of the proximal celiac axis caused by extrinsic compression of the median arcuate ligament (Figure 2A). Compression was more intense on expiration. Poststenotic dilation was also observed in all patients. 

Four patients (66.7%) were ASA I and 2 ASA II (33.3%).

Surgical procedure was uneventful in all patients. Operative time ranged from 65-120 min, with an average of 93±22 min. No patient had previous upper abdominal operation. Conversion to open operation did not occur. No operative complication was recorded. All patients were discharged from the hospital within 24 h after the procedure. The only postoperative complication was urinary retention that occurred in a male. The patient was effectively treated with urinary catheterization. No patient was readmitted to the hospital.

At three-month follow-up, all patients were asymptomatic. One referred two episodes of mild abdominal pain in the first two months, but he became asymptomatic afterwards. All four patients who referredweight loss prior to the operation, recovered most of the weight. CT angiography and/or MR angiography obtained at that time was normal, with no celiac axis stenosis (Figure 2B).

## DISCUSSION

The median arcuate ligament is a band of fibrous tissue that joins the left and right crura of the diaphragm to form the anterior surface of the aortic hiatusat the level of the 12^th^ thoracic vertebra^2^. The median arcuate ligament usually comes into contact with the aorta above the origin of the celiac axis. However, in some individuals, this ligament may be abnormally low and passes in front of the celiac axis, causing its compression, which is named MALS.

The treatment of MALS consists ofreleasingthe compression of the celiac axis by sectioning the median arcuate ligament^5^ ^,^ ^14^. The objective is to restore adequate blood flow through the compressed celiac trunk artery. Adequate medium arcuate ligament sectioning may be documented with intraoperative ultrasonography demonstrating the return of the blood flow to normal after sectioning of the ligament^12^.

Some authors have suggested that addition of neurolysis is also fundamental to treat the pain associated with MALS^11^. It has been hypothesized that the pain may also have a neuropathic component due to a chronic compression and/or overstimulation of the celiac ganglion^11^. Neurolysis with complete excision of the celiac nerve plexus may correct the neuropathic component of the pathogenesis of the syndrome.

Surgical treatment may be performed via laparotomy, laparoscopy, or robotic-assisted laparoscopy^2^ ^,^ ^18^. Laparoscopic decompression of the celiac trunk has become the standard treatment of MALS. Laparoscopic treatment of MALS compared with open operation has several advantages, including less morbidity, less postoperative pain, shorter recovery period, less adhesions, less blood loss, faster return to normal activities, and better cosmetic results^2^ ^,^ ^11^. 

Robotic-assisted approach has also been successfully employed to treat MALS^10^ ^,^ ^18^. However, due to cost limitation, the experience is still limited, and its use has been restricted to fewermedical centers.

In a literature review, Jimenez *et al*.^9^ analyzed postoperative outcome of 400 patients subjected to surgical treatment of MALS between 1963 and 2012. Eighty-five percent (339/400) of patients had immediate postoperative relief of symptoms and 6.5% (26/279) had symptom recurrence. The incidence of complications was 11.6% for laparoscopic approach and 6.5% for open surgery. The most common complications of laparoscopic approach were bleeding and pneumothorax, and of open surgery were thrombophlebitis, stroke, and gastroesophageal reflux. Laparoscopy conversion to open surgery occurred in 11 of 121 (9.1%)patients due to bleeding. There were no procedure-related deaths in both approaches.Gastroesophageal reflux disease may develop after surgical treatment of MALS, either after open surgery or laparoscopic approach^5^ ^,^ ^9^. This is possibly due to inadvertent dissection of the esophageal hiatus. In case of opening of the esophageal hiatus, it should be properly closed^12^.

Diagnosis of MALS may be difficult. Typical symptoms of MALS include recurring epigastric pain, mainly postprandial, nauseas and vomiting, reduced appetite and weight loss^11^.However, an expressive number of patients with MALS do not referrer these typical symptoms. In our series, one third of the patients did not complain of neither postprandial pain nor weight loss.In addition, the typical symptoms may mimic other diseases, such as peptic ulcer and cholelithiasis. Therefore, complete medical evaluation, including laboratory exams, endoscopy and ultrasonography should be performed to exclude other medical conditions.

Several authors have confirmed that adequate selection of patients is the most important factor to improve surgical treatment outcome^9^ ^,^ ^22^.However, selecting patients who are likely to benefit from surgery is a challenge. As in our series, combination of extensive medical evaluation to exclude other diseases and typical findings of high grade of celiac trunk stenosis on angiographyis of paramount importance to diagnose MALS.

Sustained symptom relief has been reported in 80% to 100% of patients with MALS who underwent surgical decompression, depending on several factors, including patients’ selection and severity of celiac trunk stenosis^5^ ^,^ ^10^ ^,^ ^19^.Roseborough^19^ reported subjective improvement of symptoms in 14 (93%) of 15 patients treated laparoscopically, with a mean follow-up period of 44.2 months.The followingclinical factors may indicatebetter prognosis: postprandial abdominal pain, age between 40 and 60 years, weight loss greater than 20 pounds, and absence of history of mental illness or alcohol abuse^22^.

## CONCLUSION

Laparoscopic treatment of MALS is safe and effective in relieving the clinical manifestations of patients.
